# The Effectiveness of Multi-Label Classification and Multi-Output Regression in Social Trait Recognition

**DOI:** 10.3390/s21124127

**Published:** 2021-06-16

**Authors:** Will Farlessyost, Kelsey-Ryan Grant, Sara R. Davis, David Feil-Seifer, Emily M. Hand

**Affiliations:** 1 Agricultural & Biological Engineering, Purdue University, West Lafayette, IN 47907, USA; wfarless@purdue.edu; 2 Computer Science, Ithaca College, Ithaca, NY 14850, USA; kgrant5@ithaca.edu; 3 Computer Science and Engineering, University of Nevada, Reno, NV 89557, USA; sarad@nevada.unr.edu (S.R.D.); dfseifer@unr.edu (D.F.-S.)

**Keywords:** first impressions, social traits, multi-label classification, multi-output regression, machine learning

## Abstract

First impressions make up an integral part of our interactions with other humans by providing an instantaneous judgment of the *trustworthiness*, *dominance* and *attractiveness* of an individual prior to engaging in any other form of interaction. Unfortunately, this can lead to unintentional bias in situations that have serious consequences, whether it be in judicial proceedings, career advancement, or politics. The ability to automatically recognize social traits presents a number of highly useful applications: from minimizing bias in social interactions to providing insight into how our own facial attributes are interpreted by others. However, while first impressions are well-studied in the field of psychology, automated methods for predicting social traits are largely non-existent. In this work, we demonstrate the feasibility of two automated approaches—multi-label classification (MLC) and multi-output regression (MOR)—for first impression recognition from faces. We demonstrate that both approaches are able to predict social traits with better than chance accuracy, but there is still significant room for improvement. We evaluate ethical concerns and detail application areas for future work in this direction.

## 1. Introduction

First impressions, while almost instantaneous, have serious and lasting consequences in spheres of life such as politics, career advancement and judicial proceedings [1]. Assumptions made about seemingly trivial facial details can impact the degree to which that person is perceived as trustworthy, dominant or attractive, and may subsequently affect whether they are taken seriously or treated fairly in their interactions with others.

Given an image of a face, people form first impressions within 100 milliseconds, which is less time than it takes to blink [2]. First impressions involve the determination of three social traits: attractiveness, dominance, and trustworthiness. These social traits have strong evolutionary advantages and have been long studied in the cognitive psychology community [3,4,5,6,7,8,9,10,11]. The study of social perception based on physical traits, a field which has existed for many years in psychology, is just now making its way into computer vision and machine learning, with only one major work to date [12]. However, [12] approaches the problem from a computational perspective, crowd-sourcing data collection and not considering the social traits universally accepted by the psychology community.

A model capable of determining social traits from faces, thus determining first impressions, would give researchers within psychology and behavior science another analysis tool at their disposal. The development of automated methods capable of predicting social traits will allow for future work in bias mitigation based on first impressions. Possible real world applications of this technology include, but are not limited to: the minimization of bias in hiring procedures, improvements in outcome prediction in political races and increased fairness in jury decisions.

Detection and classification of human facial attributes (describable features of faces) using computer vision and machine learning is a well-studied and rapidly-advancing field [13,14,15,16,17,18,19,20]. Convolutional Neural Networks (CNNs) are predominantly used for these learning tasks. CNNs have been trained to recognize a wide range of semantic features including gender, hair color, expression, and ethnicity [14]. These attributes are relied on by humans, both consciously and subconsciously in developing first impressions of one another.

This work seeks to be the first to address the following question, integrating psychology and computer science research: can first impressions be reliably identified from face images using automated methods? We introduce two models, a multi-label classification (MLC) model and a multi-output regression (MOR) model capable of recognizing *dominance*, *trustworthiness* and *attractiveness*—the three dimensions of first impressions—from face images. Our experiments demonstrate the effectiveness of both methods. Additionally, we detail the ethical considerations of our work and propose directions for future work. Our extensive experiments demonstrate the effectiveness of both methods.

## 2. Related Work

The detection of social traits from images and video is a relatively untouched field. We detail the related field of facial attribute recognition, along with prior research in first impression understanding from psychology and the limited work available in automated social trait recognition.

### 2.1. Facial Attributes

Facial attributes are describable features of faces and have been studied for over a decade under this name. Prior to the introduction of facial attributes, age [21], gender [22] and race [23] recognition were popular problems in face processing. Extensive publications within the field of facial attribute recognition date back over a decade. Ref. [13] introduced the first search engine capable of searching for faces based on human-describable facial features. In [14], the authors present a model for both binary detection of facial attributes as well as a model for learning similarity between two faces. Ref. [17] investigates the usage of multi-task learning for simultaneous detection of facial landmarks and attributes. Most recent works focus on the problem of recognizing facial attributes in unconstrained images and video [13,14,24,25,26,27,28]. With the introduction of a large-scale benchmark dataset—CelebA—and the use of deep CNNs for attribute recognition, impressive improvements have been made in the last few years [18]. However, most methods use external data for training, typically from large-scale datasets not labeled with facial attributes [29,30]. State-of-the-art methods employ deep CNNs, with a focus on using smaller models and training directly from attribute data [19,31,32,33]. [34] provides a thorough review of facial attribute recognition since its inception. Facial attributes are directly related to social traits and first impressions, as has been shown in the psychology research on the topic.

### 2.2. First Impressions in Psychology

There have been decades of research on first impressions from faces in the field of psychology, with the first work in 1954 [35]. It has been shown that with very brief exposure to a face stimulus, people are capable of forming first impressions of faces within 100 ms [2], and there is strong agreement in these judgments, even across different cultures [1]. However, the research on the accuracy of first impressions is mixed, with some saying that first impressions are generally correct [36], and others saying that they are mostly incorrect [37]. Whether or not the first impressions we form are correct, they have real-life consequences. It has been shown that first impressions affect the way we vote in political elections [38]. First impressions of dominance and maturity of a company’s managing partners are directly correlated with financial success [39]. Baby-faceness—facial traits associated with a lack of dominance and increased trustworthiness—and attractiveness impacted the ruling of judges in small claims court [40]. Extensive psychology research exists examining social bias resulting from first impressions of facial attributes [38,41,42,43,44], yet there are relatively few forays into this field that make use of computer vision. With first impressions having such high real-world impact, it is important to study this problem from a computational perspective. If we can automatically recognize these social traits from images and video, then we can more easily recognize our biases and manage them.

### 2.3. Social Traits

Papers in the field of automated social trait recognition are severely limited. In [8], the authors used distances between 179 facial landmarks as well as color information for the features in their social trait classifier. Their work focused on recognizing social traits in “ambient”—unconstrained—images. They use a single layer neural network as their classifier, with the facial distances and color as input features, and social traits—trustworthiness, attractiveness, and dominance—as the output. They reversed the process and were able to generate typical facial features associated with different social traits and visualize the results as cartoon faces. The face images in [8] were never publicly released, so we are unable to determine the conditions in which the “ambient” face images were collected, e.g., lighting and pose variations. Because of this, the work is not reproducible.

In [12], the authors aim to recognize social features from images using deep learning. They apply a regression-based CNN to the problem of recognizing four traits—trustworthiness, dominance, IQ, and age. They used crowd-sourcing to collect data for this problem, and tested several popular deep architectures including VGG-16, VGG-19 [45], and MOON [29]. Unlike [12], the proposed work focuses on the three social traits recognized in the psychology community as the factors for first impressions—trustworthiness, dominance, and attractiveness. The proposed work takes advantage of other datasets collected for this problem, specifically those collected by researchers in psychology.

There were research challenges in the 2016 European Conference on Computer Vision and the 2017 Conference on Computer Vision and Pattern Recognition to recognize first impressions from Vlogger videos, using both audio and visual data. However, the first impressions in this challenge did not consist of the three social traits accepted in psychology, but rather the Big-5 personality traits—extraversion, agreeableness, conscientiousness, neuroticism, and openness. Only one group was able to outperform the baseline—using CNNs and an ensemble of decision trees—by less than 1% on average [46].

The Karolinska Directed Emotional Faces (KDEF) dataset is the only available dataset with real-value image labels for *attractiveness*, *dominance*, and *trustworthiness*. This labeling scheme is discussed in [47], where the authors examine the variability of first impressions due to factors such as the emotional expression of the face, as well as the angle at which the face is being viewed. The study asked individuals to rank photos taken from the KDEF database on *trustworthiness*, *dominance*, and *attractiveness* with 1 being “not at all” and 7 being “very much” [47]. The authors found that not only was there a significant interaction between emotional expression and rankings of social judgment, but that this interaction varied across social attribute types. When viewpoint was considered in conjunction with emotional expression, there was significant interaction with several combinations resulting in outliers [47]. This interaction between emotional expression and social trait presentation underscores the importance of using at least one dataset in training and testing both our MLC and MOR algorithms that included different emotional expressions as well as different viewing angles.

A key component of our study is the incorporation of the Basel Face Database (BFD) into a large compilation dataset, which enables us to take advantage of pre-training to improve the accuracy of the multi-output regression algorithm. In [48], the authors document the collection and verification of a new facial dataset known as the Basel Face Database. Based on 40 facial identities, the BFD is composed of the manipulation of the photographs by altering what are known as the Big Two and Big Five personality dimensions. The Big Two consists of agency and communion, and the Big Five consists of openness to experience, conscientiousness, extraversion, agreeableness, and neuroticism. The authors successfully manipulate these seven personality dimensions such that surveyed participants were able to detect the direction of changes made along the face space [48]. The extreme similarity between *dominance* and agency as well as *trustworthiness* and communion allows this dataset to be used alongside the KDEF dataset despite the dissimilarity in social trait labeling.

In this work, we introduce two models for social trait recognition from face images: a multi-label classification (MLC) model and a multi-output regression (MOR) model [49]. These models differ from [8] and [12] by employing a network with multiple outputs rather than a single trait prediction. Additionally, we make use of a much more lightweight network architecture than those used in [12].

## 3. Methods

In this section, we detail the proposed methods. We discuss the data used, data cleaning methods, the construction of the compilation dataset, and finally the MLC and MOR models.

### 3.1. Data

**Figure 1:**
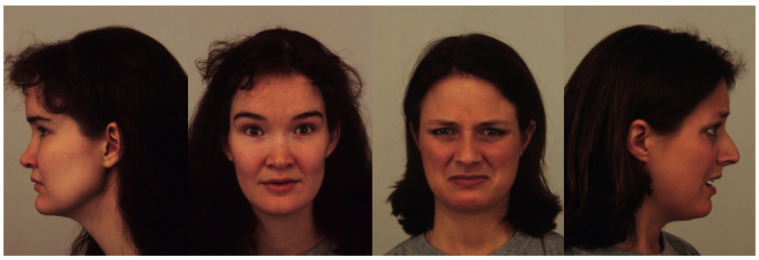
Example images taken from the KDEF dataset [47].

**Figure 2:**
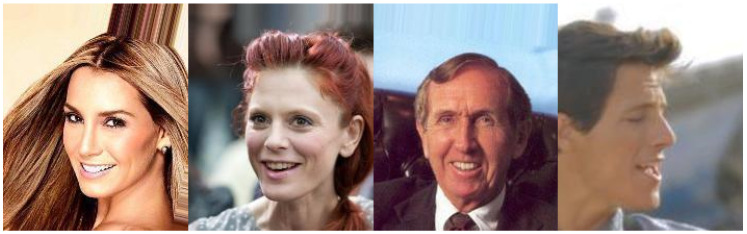
Example images taken from the CelebA dataset [18].

**Figure 3:**
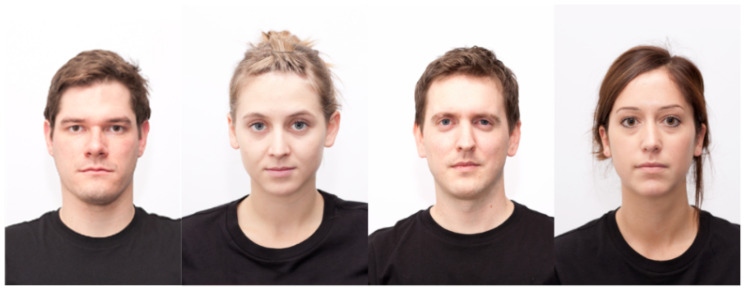
Example images taken from the BFD dataset [48].

**Figure 4:**
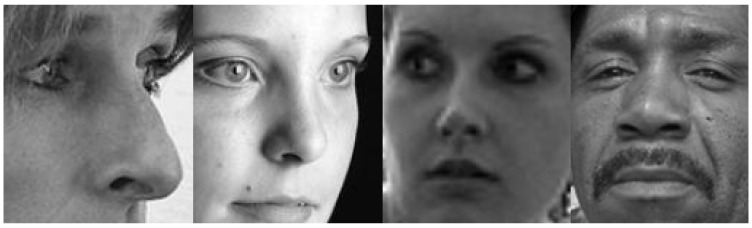
Example images taken from the AFLW dataset [12].

Our study makes use of four datasets of varying sizes with different viewing angles, color scales, and labeling schemes. Figure 1, Figure 2, Figure 3 and Figure 4 show examples from the four different datasets: KDEF, CelebA, BFD and AFLW, respectively. From these examples, it is clear that the datasets vary significantly. Both KDEF and BFD were collected in a lab setting with consistent lighting and participant positions. BFD in particular consists of only frontal faces while KDEF consists of frontal, off-frontal and profile faces. Both CelebA and AFLW consist of images taken in unconstrained environments. Working with AFLW is particularly challenging since the images are grayscale and many faces are cropped very closely, meaning some facial features may be missing. The key features of each dataset are detailed in Table 1.

### 3.2. Data Cleaning

We face the issue of inconsistent poses, lighting, and cropping in the AFLW dataset. Some photos capture only one or two features of an individual (mouth and nose, nose and eyes, etc.). Given that our research aims to determine the social traits associated with multiple features of the face at once, using images that do not satisfy this criterion poses a risk to the accuracy of the models. The AFLW dataset consists of about 6300 faces, so the process of manually determining if a photo is usable is time-consuming. To combat this, we use OpenCV [50] and Dlib [51] to differentiate the practical photos from the unusable images. We detail this process below.

OpenCV and Dlib are used to identify 68 facial landmarks in each of the 6300 images in AFLW. If all 68 landmarks are not detected, it is likely that the whole face is not present in the photo or that the face has a very challenging pose and these photos are removed from consideration. Additionally, if two faces are detected in the same image, then that image is also removed, as all of the images in AFLW contain only a single individual.

After applying the above algorithm to remove non-viable face images, 2009 images remain from AFLW. After this considerable downsize, we then begin the task of manually categorizing the images as “full-frontal”, “off-frontal”, and “unusable”. A “full-frontal” photo is defined by full and even exposure of the entire face with all necessary features. An “off-frontal” photo is defined as any head position that gives more emphasis to some features over others. An “unusable” photo is any photo that has a key feature obstructed, whether it be hair covering an eye or a hand placed over the mouth. Dividing the images into these categories allows us to filter out images with flaws that are not initially detected by our algorithm, and organize the remaining images for the testing and training of our network. This resulted in 1092 “full-frontal” images, 869 “off-frontal” images, and 59 “unusable” images from AFLW.

### 3.3. Building A Compilation Dataset

To date, KDEF is the only dataset labeled with the three social traits that make up first impressions. Even the full KDEF dataset is not labeled with these traits, only 1100 images. Rather than simply training a model on these KDEF images, we aim to take advantage of multiple sources of data, each labeled with a subset of social traits. We combine these sources of data into a new compilation dataset consisting of AFLW, BFD and CelebA. Only KDEF contains labels for all three social traits—*attractiveness*, *dominance* and *trustworthiness*—and so it is reserved for fine-tuning and evaluation. AFLW, BFD and CelebA each have labels for a subset of these traits, and the labels for each dataset vary significantly (see Table 1). Due to the dissimilarity in labeling between the datasets, it is necessary to implement a labeling scheme that only uses image labels of *attractiveness*, *dominance*, and *trustworthiness*. If a label is not available in a dataset for one of the social traits (such as dominance in CelebA), then that social trait is ignored for that set of data. Only *attractiveness* is used for CelebA, and *trustworthiness* and *dominance* for AFLW. BFD contains labels for the Big Two personality dimensions communion and agency, which have a direct relationship to *trustworthiness* and *dominance*, respectively, and so these two traits are used for BFD [48].

All three social trait labels in the compilation dataset need to be normalized to the range of 1–7. CelebA’s binary labels are mapped from {0,1} to {1,7}, so 0 labels are assigned a value of 1 in the compilation dataset and 1 labels are assigned a value of 7. We map the labels in AFLW from 0 to 2 to 1–7 using $l=3l^{\prime }+1$. Here, *l* is the new label and $l^{\prime }$ is the original AFLW label. We map the labels in BFD from 1 to 5 to 1–7 using $l=\left(l^{\prime }-1\right)\cdot \frac{3}{2}+1$. Again, *l* is the label for the new compilation dataset and $l^{\prime }$ is the label from the original BFD. We note that the labels in AFLW, BFD and KDEF are real values, not categories.

### 3.4. Multi-Label Classification

**Figure 5:**
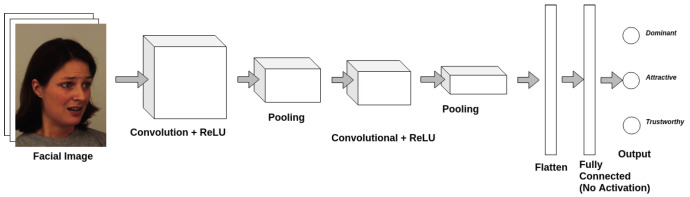
General CNN architecture used to learn the three social traits of interest. The architecture is implemented in TensorFlow [52].

We introduce a small multi-task CNN for binary multi-label classification of social traits. The architecture for the CNN is shown in Figure 5. The CNN has only two convolutional layers, two pooling layers and a fully connected layer for feature extraction. A ReLU activation is applied at both convolution layers. We use a sigmoid cross entropy loss for the MLC model. Rather than utilizing a large-scale state-of-the-art model, we choose to use a small custom model for this problem. Our prior work shows that these small models perform better on these subtle facial feature recognition problems and in settings with small, noisy datasets [19,31,32,52,53]. We start with classification, rather than regression to see if our model is capable of learning binary labels for social traits. As a baseline, we train our MLC model on KDEF, mapping the real-value labels from KDEF to binary labels using simple thresholding.

With KDEF having roughly 1000 images labeled with social traits, over-fitting, or simply not having enough data to learn are major concerns. Therefore we also pre-train a model on CelebA for attribute recognition and use transfer learning to predict binary social traits in KDEF. By training the network to recognize facial attributes on the larger CelebA dataset, when training on KDEF, ideally the network would make use of the previously learning facial attributes that play a role in social trait recognition. Generally, this increases the effectiveness of training on small datasets, decreasing discrepancies in test accuracy and smoothing the steady state loss behavior [54]. The MLC model is trained until convergence on CelebA (indicated by a plateau in validation accuracy) and then fine-tuned on KDEF.

### 3.5. Multi-Output Regression

Rather than over-simplify the problem of social trait recognition as a binary problem, we investigate the feasibility of predicting real values for these social traits. We introduce a multi-output regression (MOR) model for this problem. The same CNN architecture from before is used (see Figure 5), but rather than use sigmoid cross-entropy loss, we utilize a Euclidean loss on the real-valued labels. The baseline is again training directly on KDEF from random initialization.

Similarly to the MLC model, we introduce transfer learning using CelebA in order to improve model accuracy and stability. We utilize the same CNN pre-trained on CelebA (with binary attributes). The network is then fine-tuned on the real-valued KDEF dataset for MOR.

Finally, the compilation dataset comes into play. The compilation dataset consists of images from CelebA, BFD and AFLW with labels all mapped to the same scale as KDEF. Rather than fine-tuning on one dataset at a time, as is typically done when training on multiple datasets, the CNN is trained on all three datasets simultaneously. This is made possible through the implementation of a custom Euclidean loss function which ignores missing labels. This allows the network to update weight and bias values only based on relevant image labels. For example, given an image from CelebA, the loss is calculated with respect to the *attractiveness* prediction and the predictions for both *dominance* and *trustworthiness* are ignored. This loss is calculated for each image in the batch and then the mean over these individual losses is taken as the batch loss.

This type of loss function could be applicable in other domains where few datasets exist for a particular set of labels or where some datasets are missing relevant labels.

## 4. Results

We use five-fold cross-validation to evaluate the effectiveness of both the MLC and MOR models. This provides us with a general overview of the performance of each model across multiple validation sets. We begin by breaking the KDEF dataset into 5 random splits of even size, then we repeatedly train on four splits and test on the fifth until all splits have been evaluated.

### 4.1. Baseline Multi-Label Classification

The baseline multi-label classification algorithm is trained for 50 epochs each fold with a learning rate of 0.001 and a batch size of 20. The baseline MLC model is trained from random initialization on KDEF, to provide a starting point for improvements through transfer learning.

**Figure 6:**
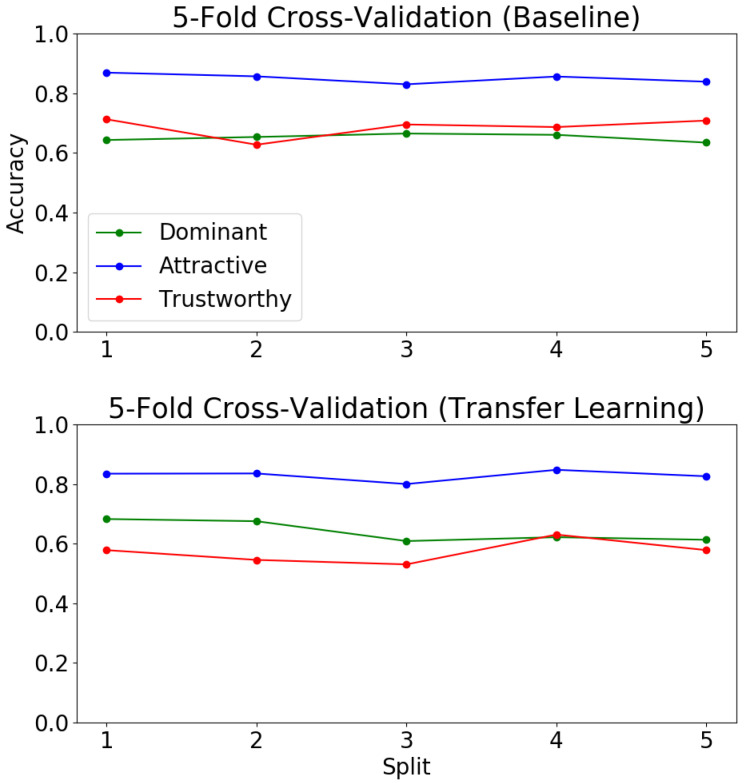
Five-fold cross-validation on baseline multi-label classification model vs. transfer learning multi-label classification model.

Figure 6 shows the results of our baseline MLC model on the five KDEF splits. We found that over all five test splits, the accuracy for *attractiveness* was consistently higher than that of *trustworthiness* and *dominance*. The correlation between perceived *attractiveness* in facial data with facial symmetry is well-studied [55]. Thus, it comes as no surprise that it is easier for a CNN to recognize the presence of *attractiveness* than it is for it to recognize *trustworthiness* or *dominance* in a face.

The accuracy for all three social traits is over 60% in all splits, suggesting that the algorithm is capable of making a binary prediction about the presence of these traits in a face with better than chance accuracy. This is impressive for a simple model trained from scratch on such a limited amount of data (880 images in each training fold).

### 4.2. Multi-Label Classification with Transfer Learning

As transfer learning tends to improve performance on many classification problems with limited training data, we decided to first pre-train our MLC model on CelebA for attribute prediction and then fine-tune it on KDEF. We pre-train the model on CelebA to predict 40 binary facial attributes for five epochs. This model is then fine-tuned on the KDEF dataset using the same five-fold cross validation from our baseline experiments. The model is fine-tuned for 50 epochs with a learning rate of 0.001 and a batch size of 20. Figure 6 shows the results of transfer learning for MLC on the KDEF dataset.

Surprisingly, transfer learning in this case resulted in a reduced accuracy across all splits on *attractiveness* and *trustworthiness*. There was, however, a slight increase in accuracy on *dominance*. This is interesting as it indicates that the pre-training task of attribute prediction is not helpful with the downstream task of social trait recognition. This is likely due to a variety of influences, including the well-documented noise in the CelebA dataset [31], as well as the limited data available for training.

### 4.3. Baseline Multi-Output Regression

To evaluate the MOR model, we again use five-fold cross validation. Whereas with the MLC, we looked at accuracy of the algorithm in predicting the three social traits of interest, here we consider the Euclidean distance as our metric for accuracy. As we are no longer working with categorical data, but rather real-valued labels, Euclidean distance provides a measurement of distance for model evaluation. This provides a measure of average distance from the predicted value to the ground truth value, but fails to consider the spread of the predictions (from 1-to-7) and, subsequently, the ability of the regression algorithm to predict outlier values.

To provide a method for comparison of the spread of predictions when analyzing each variation of the algorithm, we use a binning metric that measures average accuracy over each of the five splits for ground truth values within each bin. The real value interval from 1-to-7 is segmented into bins of size 0.5. Predictions are compared to their respective ground truth values and, if they fall within a 0.5 radius of the ground truth, it is considered a correct prediction for the bin which the ground truth occupied.

**Figure 7:**
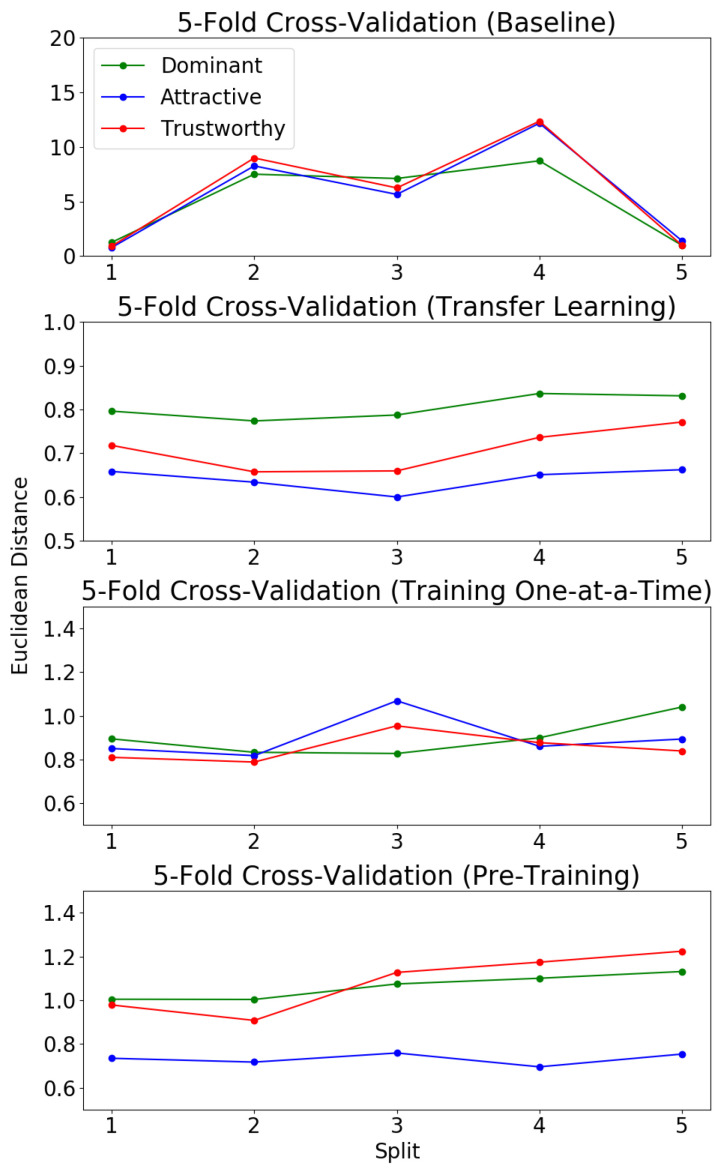
Five-fold cross-validation on each regression model trained over 50 epochs.

The baseline MOR algorithm is trained for 50 epochs on each fold with a learning rate of 0.01 and a batch size of 20. Figure 7 shows the results of the baseline MOR model (trained from scratch on KDEF). The baseline model’s high average Euclidean distance for 4 out of 5 splits might be attributed to two factors. First, the labels for *attractiveness*, *dominance* and *trustworthiness* for the KDEF dataset are heavily concentrated around 2.5–3.5. The lack of images labeled with values closer to the outliers, 1 and 7, means that the network is limited in its ability to learn and predict similar outlier values. Second, the small size of the dataset makes network over-fitting to this data likely. This might lead to the large Euclidean distances seen when applied to the test splits since the network was no longer adequately generalized for the total dataset, but rather had learned the “random regularity” contained within the training splits [56].

**Figure 8:**
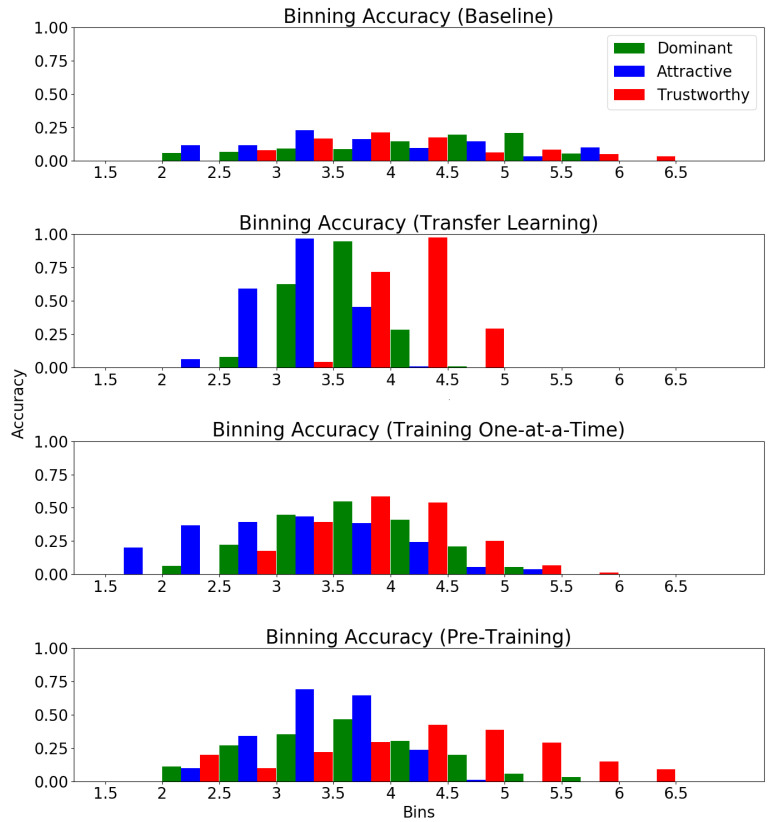
Average binning accuracy over 5 splits for each regression model.

The binning accuracy of the baseline MOR model (shown in Figure 8) is fairly well distributed across all bins. This shows that while the predictions made by the baseline MOR model are poor, they are not limited to a specific region of label values. This is to be expected as the MOR baseline model has no pre-training, and a regression problem is much more challenging than classification.

### 4.4. Multi-Output Regression with Transfer Learning

After pre-training the network on the 40 facial attributes in CelebA for five epochs, the network is then fine-tuned on the KDEF dataset training folds as a MOR algorithm for 50 epochs each fold with a reduced learning rate of 0.001 and a batch size of 20.

Figure 7 shows that transfer learning significantly reduces the average Euclidean distance across all five KDEF folds. While the five-fold cross-validation of the baseline model resulted in low binning accuracies with a Gaussian distribution with a high standard deviation, we see that the transfer learning model follows a similar Gaussian distribution, but with a much lower standard deviation and higher binning accuracies (Figure 8). This standard deviation is so low that, for all three social traits, the model was able to make no correct predictions of outlier values. Unlike the behavior of the baseline model, the transfer learning model appeared unable to learn the variation within the data and instead over-generalized to predict a value that would minimize the loss regardless of the image label. While this results in a lower average Euclidean distance between folds than that of the baseline model, it means that the model is only capable of making accurate predictions of ground truth values that show up with a high frequency in the training splits.

### 4.5. Multi-Output Regression with Serial Learning

In this and the next section, we discuss our experiments and subsequent results using the compilation dataset. First, we detail a comparison with serial learning. That is, fine-tuning on each dataset one at a time, rather than all together.

The network architecture shown in Figure 5 was first trained for 20 epochs as a multi-output regression algorithm on CelebA, at which point the loss converged, with a batch size of 300 and a learning rate of 0.01. This pre-trained model was then fine-tuned on the AFLW and BFD datasets sequentially for 20 epochs each until loss had converged in both instances. When training on the AFLW dataset, a batch size of 20 was used, and when training on the BFD dataset, a batch size of 10 was used. In both cases, the learning rate was 0.01.

Compared to the baseline MOR model, the serially trained network demonstrated a much lower Euclidean distance across all five splits (see Figure 7). Furthermore, the standard deviation of the binning accuracies, while still not as well distributed as the baseline MOR, showed a major improvement over that of the transfer learning model (see Figure 8). This likely means that the under-fitting of the network, which occurred with the transfer learning model, was avoided since the model was required to learn fewer attributes.

### 4.6. Multi-Output Regression with Parallel Learning

The network architecture shown in Figure 5 was trained for 20 epochs as a MOR algorithm on the compilation dataset until the loss converged. This pre-trained model was then fine-tuned for an additional 50 epochs on each fold of the KDEF dataset as a multi-output regression algorithm with a learning rate of 0.001 and a batch size of 20.

Both the serially training and parallel trained models resulted in an average Euclidean distance for *attractiveness*, which was lower than that of *dominance* and *trustworthiness*. This finding, along with the results seen in the MLC model five-fold validation where *attractiveness* was predicted with the highest accuracy, suggests that *attractiveness* may be an easier social trait for a CNN to learn and predict from facial data than *trustworthiness* and *dominance*, which both had higher average Euclidean distances across testing splits (see Figure 7).

The parallel-trained model also showed substantial improvements versus the baseline model when considering Euclidean distance. All five of the testing splits resulted in Euclidean distances that, while still slightly greater than those of the transfer learning model, were significantly less than the baseline Euclidean distances. Furthermore, the parallel-trained model was able to not compromise the high standard deviation of the average binning accuracy seen in the baseline model while simultaneously increasing average binning accuracy in all three social traits.

## 5. Ethical Considerations

With any work involving face processing, there are ethical questions that arise. In particular, with this work, there are important ethical considerations which must be addressed. Physiognomy is the pseudoscience of assessing a person’s character from their face or physical appearance and is a very old practice dating back to ancient Greek philosophers [57]. While it has been shown that social traits and therefore first impressions can be judged from faces with high agree-ability, it does not mean that these judgements truly align with an individual’s character. These social traits have strong evolutionary advantages and have been long studied in the cognitive psychology community. Attractiveness is linked to fertility and healthy offspring, and so is essential in finding a mate [3]. Symmetry is a strong cue for attractiveness [4], but what is judged as attractive varies greatly between cultures [5]. A person’s attractiveness tends to affect our judgments of them in general, so much so that this effect has been dubbed the “attractiveness halo” and has been studied extensively [6]. Dominance (also referred to as competence in the literature) is a way of judging intelligence and ability, which is important when determining the roles of people in a tribe or community [7]. Dominance is associated with a wider jaw, and more masculine features [8]. People with baby faces are considered less competent than others [9]. This makes intuitive sense, as youth is associated with a lack of experience. Quickly judging trustworthiness allows for an immediate assessment of danger [10]. Trustworthiness is linked to smiling and happy faces, with frowning faces indicating a lack of approachability [11]. All three dimensions of first impressions are highly related and introduce strong biases into our perception of people.

Though first impressions have some evolutionary basis, they can often be incorrect, having significant impacts on our lives. First impressions of dominance have influence over political elections [58] and hiring [59]. Positive first impressions allow us to give people the benefit of the doubt in future interactions [60]. These so-called primacy effects can also have the opposite impact, with negative first impressions leading to negative assumptions in future interactions [61]. If we can better understand how these judgments are made, then we can control for these biases. When we are aware of our biases, we are much better at properly managing them [60]. In this work, we seek to address the first part of this, automatically recognizing social traits from images. Rather than arguing that we are capable of recognizing character traits from faces, we are instead building a system that is capable of identifying human ratings of first impressions. From here, the next steps will involve evaluating physical features as they relate to these social traits. Further work can then be done in developing implicit bias training using these systems as a guiding framework.

## 6. Conclusions

In this paper, we introduce a multi-label classification algorithm and multi-output regression algorithm to identify social traits from facial data. We sought to improve upon these models using various methods: transfer learning, serial learning, and parallel learning.

We successfully demonstrated the ability of this multi-label classification algorithm, implemented using a shallow, four-layer CNN, to make a binary prediction of the presence of three social traits (*attractiveness*, *dominance*, and *trustworthiness*) in facial data. We attempted to improve upon the performance of the model by transfer learning using a larger dataset with dissimilar labels, but were unable to achieve superior results compared to the baseline multi-label classification algorithm.

The multi-output regression algorithm predicted a real-value signifying the degree to which one of the three previously mentioned social traits was present in an image. Transfer learning using a larger dataset resulted in a significantly smaller Euclidean distance between predicted values and the ground truth for MOR. However, this was only achievable for a small band of label values. Serially pre-training on multiple datasets allowed us to increase this band of label values, but resulted in a higher average Euclidean distance. Our greatest success occurred when pre-training the network in parallel on a larger compilation dataset of dissimilar images. This resulted in the largest band of predicted values with an acceptably low average Euclidean distance.

We showed that it is possible for both MLC and MOR models to accurately predict social traits from face images. While there are improvements to be made, automated methods capable of predicting social traits will have significant impacts in many areas of life including the minimization of social bias in social interactions, such as hiring practices, as well as better understanding the role these biases may play in our lives. This work is the first in the direction of automatically recognizing first impressions from images using the well-established social traits from psychology: *attractiveness*, *dominance*, and *trustworthiness*. Future work should focus on collecting more data for this problem, investigating ethical concerns, linking facial attributes and social traits and developing implicit bias training using the results of such work.

## Figures and Tables

**Table 1:** Comparison of the key features and image labels of the AFLW, BFD, CelebA, and KDEF datasets.

Dataset	Number of Images	Key Features	Labels (Type)
AFLW	6300	Grayscale, all ages and races, tightly cropped inconsistent viewing angles	4 (Real value 1–4)
BFD	40	Full color, Caucasian adults with neutral expressions, full frontal	2 (Real value 1–5)
CelebA	200,000	Full color, celebrities, inconsistent viewing angles	40 (Binary)
KDEF	1152	Full color, Caucasian adults making ranges of different facial expressions, full frontal	3 (Real value 1–7)

## Data Availability

We use four publicly available datasets in this work: CelebA [18], BFD [48], KDEF [47] and AFLW [12]. CelebA: http://mmlab.ie.cuhk.edu.hk/projects/CelebA.html (accessed on 1 June 2019); BFD: https://bfd.unibas.ch/en/ (accessed on 1 June 2019); KDEF: https://www.kdef.se (accessed on 1 June 2019); AFLW with first impressions: https://github.com/mel-2445/Predicting-First-Impressions (accessed on 1 June 2019).

## Abbreviations

The following abbreviations are used in this manuscript:

CNN	Convolutional Neural Network
MLC	Multi-Label Classification
MOR	Multi-Output Regression
